# 

**Published:** 1905-05-20

**Authors:** 


### NOTICE TO CORRESPONDENTS.

All MSS., Letters, Books for Review, and other matters intended for the Editor should be addressed The Editor, "The Hospital," The Hospital Buildings, 28 & 29 Southampton Street, Strand, W.C.

The Editor cannot undertake to return rejected MS., even when accompanied by stamped directed envelope.

**Notice to Advertisers and Subscribers.**

All Advertisements, Orders for Copies of The Hospital, and Business Communications should be addressed to **THE MANAGER (Not to the Editor),** The Hospital, 28 & 29 Southampton Street, Strand, London, W.C.

The Cost of Subscription, post free, is at follows :—Per week, 3½d.; per quarter, 3s. 3d.; per half-year, 6s. 6d.; per annum, 13s. Foreign and Colonial :—Per annum, 19s. 6d., payable, in all cases, in advance.

**NOTICE.—Vol. XXXVII. of "The Hospital" October 1904 to March 1905), in handsome cloth binding, will shortly be on sale at the office of this Journal, price 10s. 6d. Cases for binding the Numbers contained in this Volume 2s. Index to Vol. XXXVII., 3½d., post free.**

**The Monthly Part for May is now ready. Price 1s., by post, 1s. 3d.**

### Medical Appointments.

**Edward Seymour Brett**, M.B., Ch.B.Edin., Assistant Medical Officer to the Mental Hospital at Seacliff, New Zealand. **T. Cherry**, M.D.Melb., Acting Director for Agriculture in Victoria, Australia. **D. Coutts**, M.B., C.M.Glasg., Assistant at Kingseat Asylum, Glasgow. **Alexander Douglas**, M.B., M.S. Edin., Port Health Officer, Oamaru, New Zealand. **Robert Charles Earle**, M.R.C.S., L.S.A., Health Officer of the Port of Wanganui, New Zealand. **Claude St. Aubyn Farrer**, L.R.C.P.&S.Edin., L.F.P.S.Glasg., has been reappointed Clinical Assistant at the Samaritan Hospital for Women. **Charles Albert Hingston**, M.D., B.Sc.Lond., M.R.C.S.Eng., Honorary Physician to the Crownhill Convalescent Home, near Plymouth. **J. E. Judson**, M.R.C.S., L.R.C.P.Lond., Honorary Assistant Medical Officer to the District Infirmary and Children's Hospital, Ashton-under-Lyne. **James Patrick Kelly**, M.B., Acting Officer of Health of the shire of Wyndham, Victoria, Australia. **Ramsay Mailer**, L.R.C.S.Edin., Medical Superintendent of the Ararat Lunatic Asylum, Victoria, Australia, *pro tem.* **Robert Haldane Makgill**, M.B.Edin., D.P.H.Cantab., Bacteriologist to the Health Department, New Zealand. **J. Hardie Neil**, M.B., Medical Practitioner under the Workers' Compensation for Accidents Act, Auckland, New Zealand. **John Alexander Newell**, B.A., M.B., B.Ch., Surgeon to H.M. Prison at Lyttelton, New Zealand. **W. H. Parkes**, M.B., Ch.M.Edin., Honorary Medical Officer to the Veterans' Home, Auckland, New Zealand. **George Henry Trenfield**, M.R.C.S., L.R.C P.Lond., *pro tem.*, Medical Officer of the Eighth District by the Bristol Board of Guardians. **Vivian F. Wall**, L.R.C.P., M.R.C.S., L.S.A., Honorary Anæsthetist to the Western Ophthalmic Hospital, Marylebone Road, W. **William Henry Waterfield**, L.R.C.P.&S.Irl., Chairman of the Children's Committee of the Devenport (Devon) Board of Guardians.

### Stepping Stones to Success

WRITE FOR NEW CATALOGUE OF NURSING MANUALS.

**HOSPITAL EXPENDITURE: The Commissariat.**
A Handbook for Matrons.
Cloth, 2/6 post free.

**HOSPITAL SISTERS AND THEIR DUTIES.**
By EVA E. LÜCKES.
Third Edition. Cloth, 2/6.

**A COMPLETE HANDBOOK OF MIDWIFERY.**
By J. K. WATSON, M.D.
143 Illustrations.
Cloth, 6/- net; 6/4 post free.

**MODERN NURSING OF CONSUMPTION.**
By Dr. JANE H. WALKER.
Crown 8vo. cloth, 1/- net; 1/1 post free.

**A HANDBOOK FOR NURSES.**
By J. K. WATSON, M.D.
NEW EDITION, with Examination Questions based on the contents of the Chapters. 5/- net; 5/4 post free.

**SURGICAL WARD WORK AND NURSING.**
By ALEXANDER MILES.
Second Edition. 3/6 net; 3/10 post free.

**ELEMENTARY PHYSIOLOGY FOR NURSES.**
By C. F. MARSHALL, M.D.
Illustrated. Cloth, 2/- post free.

**ELEMENTARY ANATOMY AND SURGERY FOR NURSES.**
By WILLIAM McADAM ECCLES.
Profusely Illustrated. Cloth, 2/6 post free.

**PRACTICAL GUIDE TO SURGICAL BANDAGING AND DRESSINGS.**

By WILLIAM JOHNSON SMITH, F.R.C.S.

Illustrated.

Suitable size for apron pocket. 2/- post free.

**THE NURSE'S PRONOUNCING DICTIONARY OF MEDICAL TERMS AND NURSING TREATMENT.**

Compiled by HONNOR MORTEN.

New & Revised Edition. Edited by MARY I. BURDETT.

2/- post free.

**HOW TO BECOME A NURSE.**
**The Nursing Profession: How and Where to Train.**
By Sir HENRY BURDETT, K.C.B.
New Edition ready Next Week Cloth, 2/- net; 2/4 post free.

**The Scientific Press, Ltd.** Publishers of Nursing Text-books, Charts, Case-books, &c., of all descriptions,

28 & 29 SOUTHAMPTON ST., STRAND, LONDON, W.C.

### The NURSING MIRROR.

**The Nurse's Own Paper.**

**Price ONE PENNY Weekly.**

Of all Booksellers and Newsagents, or posted direct from the Office.

*Write for Subscription Rates to The Manager,*

THE NURSING MIRROR, The Hospital Building, 28 & 29 Southampton Street, Strand, London, W.C.

